# New Hampshire

**Published:** 1897-08

**Authors:** 


*New Hampshire.*—The New Hampshire Cattle Commission will experiment on ten thoroughbred Holstein cattle that have responded to the tuberculin-test: one bull, and nine cows, all under four years of age. The milk will be fed to pigs, and the latter slaughtered and carefully examined. Six of the cows have been served, and their calves will be reared on milk from cows tested and pronounced free from tuberculosis. The tuberculin-test will be repeated once in four months.

